# Sex-Based Differences in Lung Cancer Incidence: A Retrospective Analysis of Two Large US-Based Cancer Databases

**DOI:** 10.3390/cancers16193244

**Published:** 2024-09-24

**Authors:** Kalyan Ratnakaram, Sai Yendamuri, Adrienne Groman, Sukumar Kalvapudi

**Affiliations:** 1 Department of Thoracic Surgery, Roswell Park Comprehensive Cancer Center, Elm and Carlton Streets, Buffalo, NY 14263, USA; kalyan.ratnakaram@roswellpark.org (K.R.); skalvapudi@utmck.edu (S.K.); 2 Department of Biostatistics and Bioinformatics, Roswell Park Comprehensive Cancer Center, Elm and Carlton Streets, Buffalo, NY 14263, USA; adrienne.groman@roswellpark.org

**Keywords:** NSCLC, lung cancer incidence, sex disparities, NLST, SEER

## Abstract

**Simple Summary:**

In recent years, researchers have begun to investigate the relation between sex and cancer. We wanted to look at the relation between sex and non-small cell lung cancer among high-risk patients to see if this relationship needs further exploration. In order to do this, we chose to look at large publicly available databases that contained information on lung cancer incidence. Our research found indications that females have an elevated risk of lung cancer incidence in comparison with males within high-risk populations. These results suggest that the difference in sex-specific cancer biomechanisms should be further investigated and explored.

**Abstract:**

**Background/Objectives:** Non-small cell lung cancer (NSCLC) has seen a relative rise in incidence among females versus males in recent years, although males still have a higher overall incidence. However, it is unclear whether this trend is consistent across all populations. Therefore, we retrospectively examined this relationship in two large high-risk clinical cohorts. **Methods:** First, we analyzed lung cancer incidence among individuals with a smoking history of over 40 pack-years in the National Lung Screening Trial (NLST). Then, we investigated the incidence of second primary NSCLC in patients who underwent lobectomy for previous stage I lung cancer using the Surveillance, Epidemiology, and End Results (SEER) database. We performed both univariate and multivariable time-to-event analyses to investigate the relationship between sex and lung cancer incidence. **Results:** In the NLST cohort (n = 37,627), females had a higher risk of developing primary NSCLC than males (HR = 1.11 [1.007–1.222], *p* = 0.035) after adjusting for age and pack-year history. In the SEER cohort (n = 19,327), females again exhibited an increased risk of developing a second primary lung cancer (HR = 1.138 [1.02–1.269], *p* = 0.021), after adjusting for age, race, grade, and histology. **Conclusions**: Our analysis reveals that females have a modestly higher lung cancer incidence than males in high-risk populations. These findings underscore the importance of further researching the underlying cellular processes that may cause sex-specific differences in lung cancer incidence.

## 1. Introduction

Lung cancer, the second most prevalent malignancy worldwide, is the leading cause of cancer-related deaths in both sexes. In the United States alone, an estimated 234,000 new cases and 125,000 deaths are projected for 2024, underscoring the significant public health challenge posed by lung cancer [1]. Cigarette smoking is recognized as the primary cause of lung cancer, with all histological subtypes linked to this habit [2]. Nonetheless, other factors also seem to influence lung cancer risk.

Epidemiological studies have consistently documented sex differences in the incidence and outcomes of lung cancer. Historically, males have exhibited a higher age-standardized incidence rate of lung cancer than females [3,4]. From 2015 to 2019, the incidence of lung cancer was 27% higher in males than in females [5]. Males were also diagnosed at a more advanced stage than females, leading to a poorer prognosis [6]. Much of this disparity is attributed to the higher smoking prevalence among males [7].

However, over the last two decades, a decline in smoking prevalence in the United States has led to a decrease in the incidence of lung cancer [8]. Since 2006, the annual incidence rates of lung cancer have decreased by 2.6% in males and 1.1% in females [5]. This has resulted in a relative increase in female lung cancer rates when compared to male rates. Recent studies have also indicated a rise in lung cancer diagnoses among young females compared to their male counterparts [9]. Nevertheless, despite these trends, lung cancer incidence remains higher in males in the overall population compared to females [10].

For our study, we chose to look at individuals at high risk of developing lung cancer. Risk factors for lung cancer are numerous and include age, history of smoking, prior diagnosis of malignant tumors, and occupational exposure to asbestos. Of these, smoking is the biggest risk factor for lung cancer [11]. Another important factor is age, as older individuals are more likely to develop lung cancer [11]. Selection criteria for all randomized controlled trials for lung cancer screening include smoking history and age to target higher risk individuals [12]. For example, the NLST required a 30 pack-year smoking history and participants aged over 55 as inclusion criteria [12]. Additionally, prior malignancy, although less studied, is associated with an increased risk of new lung cancer, as highlighted by the National Comprehensive Cancer Network (NCCN) and the American Association for Thoracic Surgery (AATS), which advocate for lower screening thresholds in patients with a history of malignancy [13].

Most previous epidemiological studies have focused on lung cancer incidence and sex-related differences in the general population, not specifically in high-risk populations. To explore the relationship between sex and lung cancer incidence in high-risk populations, we retrospectively performed time-to-event analyses of two publicly available clinical cohorts: the National Lung Screening Trial (NLST) and the Surveillance, Epidemiology, and End Results (SEER) databases. This information is necessary in determining whether additional research into the sex-specific physiological mechanisms that relate to lung cancer is worthwhile.

## 2. Materials and Methods

We conducted a retrospective analysis to explore the association between sex and lung cancer incidence, utilizing data from two sizable clinical cohorts: the National Lung Screening Trial database and the Surveillance, Epidemiology, and End Results database.

### 2.1. NLST Analysis

The NLST was a randomized controlled trial sponsored by the U.S. National Cancer Institute (NCI) aimed at assessing the efficacy of low-dose helical computed tomography (CT) screening in reducing lung cancer mortality among individuals at high risk [11] We used this publicly available database to examine sex-based disparities in primary lung cancer incidence.

Patient recruitment for the NLST occurred between August 2002 and April 2004. Demographic information was gathered, and participants were monitored for lung cancer occurrence throughout the trial. The NLST inclusion criteria comprised a smoking history of ≥30 pack-years, cessation of smoking within ≤15 years for former smokers, absence of prior lung cancer or other life-threatening cancers within the preceding five years, and no symptoms indicative of lung cancer such as hemoptysis or unexplained weight loss [14]. This initial screening resulted in the enrollment of 53,354 eligible participants aged 55 to 74.

**Figure 1:**
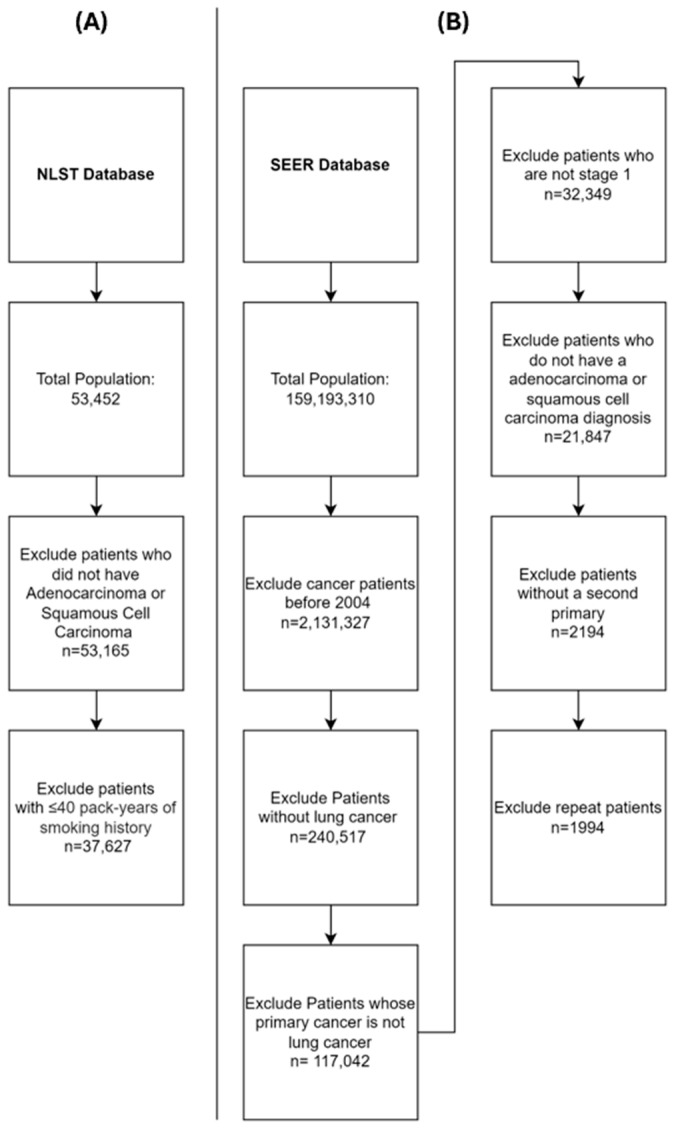
Study Selection Criteria. (**A**)—selection criteria for the NLST database. (**B**)—selection criteria for the SEER database.

For our investigation, we focused solely on patients with a smoking history exceeding 40 pack-years and those diagnosed with non-small cell lung carcinoma (NSCLC), leading to a final cohort of 37,627 individuals. We limited our patient population to those with a 40-pack-year smoking history to emulate high-risk patients and to ensure a higher incidence rate of lung cancer for the time-to-event analysis. Case selection strategy for the NLST cohort is given in Figure 1A. Details including patient age, sex, race, smoking behavior, pack-year history, and lung cancer incidence were collected from the NLST database. To conduct time-to-event analysis, the start date was defined as the date of enrollment in the trial, extending until either the last follow-up date or the date of primary lung cancer development.

The database was accessed on 11 August 2023 at the url: https://cdas.cancer.gov/nlst/.

### 2.2. SEER Analysis

The SEER Program database, overseen by the National Cancer Institute (NCI), encompasses 18 cancer registries, covering approximately 48% of the U.S. population [15]. This comprehensive database contains information regarding patient demographics, primary tumor characteristics, and follow-up data, including survival outcomes.

To identify individuals at high risk for developing lung cancer, we chose people with treated primary lung cancer and followed them to see if they developed a second lung cancer. We therefore looked at patients diagnosed with primary lung cancer between 2004 and 2020 within the SEER database. Patients with a history of any other malignancy were excluded as well as histologies other than adenocarcinoma or squamous cell carcinoma. We wanted to select patients who developed a second primary lung cancer and not recurrence of lung cancer. Subsequently, we refined our cohort to include only those with stage I disease who underwent lobectomy. We specifically selected patients with prior stage I NSCLC who underwent lobectomies, as stage I NSCLC generally has a lower recurrence rate than more advanced stages. Lobectomies are also regarded as the most effective treatment for early-stage non-small cell lung cancer [16]. Furthermore, we concentrated exclusively on these two histological subtypes, which represent the most prevalent forms of NSCLC, accounting for approximately 70% of all lung cancer cases [17]. This approach also ensured consistency between the two databases. The selection process yielded a final cohort comprising 19,327 patients (Figure 1B). Data regarding patient age, sex, race, grade of first primary lung cancer, and histology of first primary lung cancer were extracted from the SEER database. Follow-up was conducted until death, the last follow-up date, or the occurrence of a second primary lung cancer. For time-to-event analysis, the date of primary lung cancer diagnosis was defined as the starting point, extending until either the occurrence of a second primary lung cancer or the date of the last follow-up.

The database was accessed on 15 May 2023 at https://seer.cancer.gov/.

### 2.3. Statistical Analysis

Continuous data were presented as means with standard deviation and categorical data as numerical values and percentages. Independent *t*-tests were used to compare group differences for continuous variables and chi-squared tests for categorical variables.

Time-to-event univariate Cox proportional hazards modeling was used to identify significant associations between various clinical characteristics and either incidence of primary lung cancer (NLST) or incidence of second primary lung cancer (SEER). Time to last follow-up and either time to incidence of lung cancer (NLST) or time to incidence of second primary (SEER) was used as the time variable. To control confounding factors, multivariate Cox proportional hazards modeling was conducted using a stepwise backward elimination method to develop the final model, with a tolerance of *p* < 0.1 at each step.

Statistical analyses were performed using the IBM SPSS statistical software version 28.0.1.0. Plotting was performed using GraphPad Prism version 10.1.2. For all analyses, *p* < 0.05 was considered statistically significant.

## 3. Results

### 3.1. NLST

A total of 37,627 patients were examined in the NLST database, with a mean age of 62 (standard deviation (SD) = 5.1). Most of the cohort was Caucasian (92%), with former and current smokers comprising an even split (50.7% former smokers, 49.3% current smokers). The overall incidence of primary lung cancer was 4.7% (1781 out of 37,627).

For the purposes of this analysis, we compared males (n = 23,662, 62.9%) to females (n = 13,965, 37.1%) in the cohort. Males tended to be slightly older (male vs. female, *p*-value; 62.2 vs. 61.8, *p* < 0.01) and were less likely to be current smokers (47.8% vs. 52%, *p* <0.01) than females, yet they had a higher pack-year history (66.9% vs. 60.6%, *p* < 0.01) (Table 1). The mean follow-up time for males was 2273 (95% CI: 2266–2280) days and for females was 2300 (95% CI: 2292–2308) days. The incidence rate of primary lung cancer per 1000 person-years was 7.57 (95% CI: 7.23–7.93) in the overall population, 7.48 (95% CI: 7.05–7.93) in males, and 7.72 (95% CI: 7.16–8.32) in females.

Subsequently, univariate time-to-event Cox proportional hazards modeling was employed to explore the associations between age, sex, and pack-years with lung cancer incidence. Increasing patient age (Hazard Ratio (HR) = 1.076 [95% Confidence Interval (CI) = 1.066–1.085], *p* < 0.01) and pack-year history (HR = 1.007 [1.006–1.009], *p* < 0.01) were predictive of decreased time to lung cancer development. Patient sex was not significantly associated with time to lung cancer (female vs. male, HR = 1.033 [0.939–1.137], *p* = 0.502). Multivariable time-to-event Cox proportional hazards modeling, incorporating age, sex, and pack-years as covariates, along with the interaction between sex and pack-years, revealed that females had a significantly higher rate of lung cancer development than males (HR = 1.11 [1.007–1.222], *p* = 0.035) after adjusting for age and pack-year history (Table 2). Age and pack-year history were also retained in the final model, but the interaction between sex and pack-years was eliminated.

### 3.2. SEER

A total of 19,327 patients with stage I NSCLC who underwent lobectomy were included from the SEER database. The mean age of the cohort was 70.5 (SD 9.8), with the majority being Caucasian (81.3%). In the overall population, 47.5% had well/moderately well-differentiated tumors, and 22.4% had poorly differentiated tumors prior to surgery. Most patients were diagnosed with adenocarcinoma (64.8%). During the follow-up period, 9.6% of the cohort (1862 out of 19,327) developed a second primary lung cancer.

Between the two groups, an equal number of males (n = 9175, 47.5%) and females (n = 10,152, 52.5%) were observed. Males were more likely to have poorly differentiated primaries (25.4% vs. 19.6%, *p* <0.01) and squamous cell carcinoma (42.7% vs. 28.3%, *p* < 0.01) prior to lobectomy compared to females (Table 3).

Univariate time-to-event Cox hazard analysis revealed that only squamous histology (HR = 1.181 [1.073–1.3], *p* <.01) and female sex (HR = 1.12 [1.022–1.228], *p* = 0.016) significantly predicted earlier onset of second primary lung cancer diagnosis. A multivariable analysis incorporating age, sex, race, grade, and histology as covariates retained only sex and histology in the final model (Table 4). The mean follow-up time for males was 51 (95% CI: 50–52) months and for females was 58 (95% CI: 57–59) months. The incidence rate of second primary cancer per 1000 person-years was 22 (95% CI: 21.1–23) in the overall population, 16 (14.9–17.1) in males, and 30.1 (28.4–31.8) in females.

## 4. Discussion

**Figure 2:**
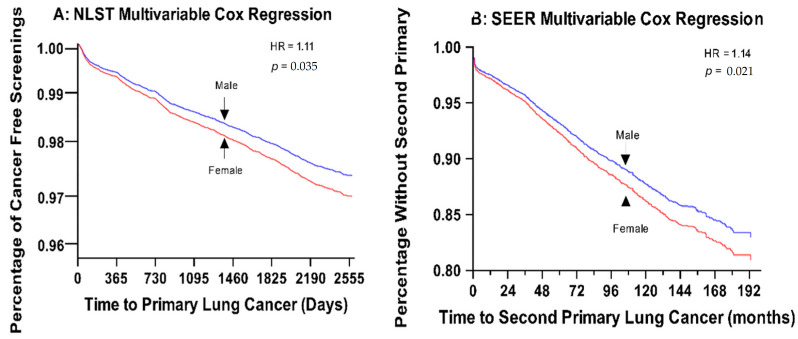
Multivariate Cox Regression Graphs for both the NLST and SEER Databases. (**A**) Multivariable cox proportional hazards model for time to primary lung cancer in the NLST database. Final model includes sex, age, and number of pack-years. (**B**) Multivariable cox proportional hazards model for second primary lung cancer in the SEER database. Final model includes sex and histopathology.

Lung cancer incidence has displayed a concerning trend in recent decades, with a consistent rise among females compared to males. Data spanning 2001–2019 show a steady decrease in male lung cancer rates, while female lung cancer rates reached a peak around 2006 before also declining, albeit at a slower pace than males [8]. Despite this, males still exhibit a higher overall incidence than females. This prompted an investigation into whether this pattern held true even within high-risk populations. The main aim of this study was to systematically compare lung cancer incidence in high-risk populations and explore sex-based differences. Our findings, based on two large publicly available databases—the NLST and SEER databases—indicate that females are at a higher risk of developing lung cancer than males, even after accounting for confounding factors such as age, smoking history, and histology (Figure 2). While previous studies have investigated sex-based disparities in lung cancer incidence, this study is potentially the first to assess sex differences in high-risk populations specifically.

The disparity in lung cancer incidence between sex can be partially attributed to differences in smoking behavior. Historically, females began smoking in larger numbers later than males and were slower to quit [7,18]. This aligns with data indicating a slower decline in lung cancer rates in females, as well as an increase in lung cancer diagnoses in young females [18,19]. To further investigate the association between gender and lung cancer incidence, as well as its relation to smoking, we examined the NLST database. Initial univariate Cox proportional time-to-event analysis did not reveal significant sex differences in lung cancer risk. However, multivariable analysis, after adjusting for the number of pack-years and age, identified female sex as an independent risk factor for lung cancer incidence. Thus, for the same number of pack-years smoked, females had an 11% higher risk of developing lung cancer than males. This finding is consistent with previous studies [20,21,22,23]. For instance, Zang et al. found that at every level of tobacco exposure, the risk of lung cancer in females was 1.2- to 1.5-fold higher than in males, suggesting that females have a higher relative risk of lung cancer than males with the same level of smoke exposure [23]. Additionally, Harris et al. found that females, compared to males, face a higher risk of lung cancer at the same level of smoking for both major forms of lung cancer, with a 70% higher risk for squamous cell carcinoma and a 50% higher risk for adenocarcinoma [21].

Well-documented differences in smoking patterns exist between males and females. Although females began smoking in larger numbers later than males and have been slower to quit, males still consume a greater overall quantity of tobacco products [24]. Consistent with this, in our selected NLST cohort, we discovered that females were more likely to be current smokers than males but had a lower pack-year history. Females tend to start smoking at a later age, smoke less frequently, inhale less deeply, and are less likely to smoke harmful non-menthol cigarettes than males. Despite reduced intensity of smoking, females exhibit a higher risk of lung cancer for a given pack-year history than males [20,23,25,26]. This suggests that a more effective metric should be implemented—perhaps pack-years is not the most accurate measure of cigarette smoke exposure when considering females. A potentially better metric, such as total duration smoked, might be a more suitable measure for screening guidelines. For instance, a 2018 study of the Norwegian population by Hansen et al. found that when smoking amount was measured as a continuous variable, female smokers had a greater risk of lung cancer than male smokers [20].

To corroborate the elevated incidence of lung cancer in high-risk females relative to high-risk males, we employed the SEER database. We examined patients with a history of prior stage I lung cancer who had undergone successful lobectomy and conducted a time-to-event analysis to ascertain the onset of a second primary. A multivariable analysis revealed that females had a 14% higher chance of developing a second primary compared to males, even after adjusting for confounders such as age, race, grade, and histology. One of the most significant risk factors for developing a second primary is continued smoking. Regrettably, data regarding smoking history were not available from the SEER database, and the absence of these data is a limitation of our study. While there is extensive data reporting on overall lung cancer incidence and sex differences, there is a scarcity of data concerning the incidence of second primary specifically. In a 2017 study of the SEER database by Thakur et al., females were found to have a higher standardized incidence ratio of second primary lung cancer than males [27]. This novel and intriguing finding that females are at a higher risk of developing a second primary warrants further investigation into the underlying risk factors.

Several potential mechanisms underpin the increased risk of lung cancer in females. One potential mechanism is estrogen-induced carcinogenesis, mediated by the altered metabolism of tobacco carcinogens [28]. Nicotine-derived nitrosamine ketones (NNKs), a group of extensively studied tobacco carcinogens, significantly contribute to the carcinogenic potential of cigarette smoke. Female sex hormones have been demonstrated to inhibit safe NNK metabolism, thereby promoting carcinogenesis [29]. Furthermore, estrogen can be metabolized into catechol estrogens through the increased activity of cytochrome P450 1B1 (CYP1B1) [30]. CYP1B1 expression is amplified by cigarette smoking, and it has been shown in animal models that female mice exposed to cigarette smoke exhibit increased CYP1B1 activation compared to male mice [31]. These catechol estrogens are subsequently activated into semiquinone and quinone intermediates, leading to the formation of harmful DNA adducts that promote mutagenesis and inhibit DNA repair [28,30,32]. However, these observed differences cannot be entirely attributed to variations in smoking patterns, as females appear to develop lung cancer at higher rates even after controlling for smoking. For instance, females who have never smoked seem to develop lung cancer at rates that exceed those of their male counterparts [33]. One explanation is that the changing patterns of lung cancer histology as well as endogenous genetic variations between the two sexes may contribute to these disparities [29].

Over the last few decades, there has been an increasing trend of adenocarcinoma compared to squamous cell carcinoma. This rising incidence of adenocarcinoma may be partially associated with increased smoking of low-tar filter cigarettes allowing for smaller particles to travel more distally within lungs. Adenocarcinoma surpassed squamous cell carcinoma as the most frequently diagnosed lung cancer in males [10]. Adenocarcinoma also exhibits a less rapid decline compared to squamous cell carcinoma in response to cigarette smoking cessation [10]. Females have been reported to carry a higher frequency of mutations in critical driver genes such as epidermal growth factor receptor (EGFR), anaplastic lymphoma kinase (ALK), Kirsten rat sarcoma (KRAS) genes, and tumor protein 53 (TP53), thereby increasing susceptibility to adenocarcinoma [8,34]. This heightened susceptibility to adenocarcinoma could account for the relative incidence of lung cancer in females compared to males. Endogenous genetic variations in females also may be responsible for increased lung cancer in females. Polymorphisms in the cytochrome P450 1A1 gene, defects in DNA repair, overexpression of the X-linked gastrin-releasing peptide receptor, and mutations in p53 have been linked to increased lung cancer pathogenesis in females [34].

Finally, exposure-related factors, including increased exposure to secondhand smoke and environmental carcinogens, may also contribute to the elevated risk in females [10]. Unfortunately, population-based quantification of secondhand smoke exposure is challenging, and there is a lack of robust data. An observational study of patients diagnosed with lung cancer in France found that 78% of nonsmoker females had environmental exposure to tobacco smoke compared to only 21% of nonsmoker males [35]. Another analysis of 37 studies found that females whose spouses smoked had a 24% increased risk of developing lung cancer [36]. Risk factors like asbestos and radon, which are known to contribute to lung carcinogenesis, have been inadequately studied in women, despite their increased exposure at home [10]. Indoor cooking fumes are another possible gender-role-dependent risk factor. One meta-analysis from Asia demonstrated that females who cook with wood or coal at home have increased odds of developing lung cancer compared to those who do not [37]. Cooking oil fumes also contain polycyclic aromatic hydrocarbons (PAHs), which are known to be carcinogenic. The effect of PAHs on lung carcinogenesis is especially seen in women from developing countries, due to improper ventilation [38]. Further investigation into these non-cigarette smoking-induced pathways of lung carcinogenesis is needed to address the disproportionate risk to females.

The limitations of our study must be acknowledged. As a retrospective analysis, our study was constrained by the data available from two databases. We could only adjust for covariates present in the datasets, and we lacked information on patients’ overall health and socioeconomic status, both of which are known to influence cancer rates. This absence may have confounded our results. In the NLST database, we used pack-years as a numerical variable, which assumes a linear relationship between pack-years and lung cancer incidence. While some studies suggest a linear dose–response between cigarette smoking and lung cancer risk [39,40,41], others indicate that this relationship may vary depending on daily consumption [42]. For those with lower daily consumption, the relationship is suggested to be logarithmic, while at higher levels of smoking, the relationship appears more linear [43]. Unfortunately, there is limited research specifically examining pack-years as opposed to cigarettes smoked per day. This presents a limitation in our study, as assuming linearity may not fully capture the complexities of smoking behavior and its impact on lung cancer risk. In the SEER database, we used patients with prior lung cancer as surrogates for high-risk individuals to observe the development of second lung cancers. This approach has limitations, as distinguishing between recurrence and new primary cancers is challenging. Additionally, the SEER database lacks data on smoking, a significant confounder in lung cancer studies. Although race was included as a covariate, the databases primarily comprised patients of Caucasian descent, which limits the study’s applicability to more diverse populations [44,45]. Furthermore, we lacked data on occupation-related lung cancer and passive smoking, both of which differentially affect males and females. This underscores the need for further research in these areas. Finally, this study’s generalizability may be limited, as both SEER and NLST are US-based.

## 5. Conclusions

Numerous epidemiological studies indicate that the incidence of lung cancer is higher in males than in females, although there has been a relative increase in lung cancer risk in females over the past few decades. However, most of these studies have concentrated on the general population. When examining high-risk patients specifically, we find that females are at a higher risk for developing lung cancer than males, even after adjusting for confounding factors such as age and smoking history. Notably, we discovered that females are at an elevated risk of developing a second primary compared to males. The increased incidence of lung cancer in females compared to males in high-risk populations necessitates a deeper understanding of the potential underlying mechanisms. Conducting further epidemiological and biological research into sex-specific differences in lung cancer incidence will lead to a better understanding of how sex plays a role in lung cancer.

## Figures and Tables

**Table 1:** Overall Patient Characteristics of the NLST Cohort as well as Differences Between Males and Females.

Variable	Overall n = 37,627	Male n = 23,662 (62.9%)	Female n = 13,965 (37.1%)	*p*-Value
Age	62 (5.1)	62.2 (5.1)	61.8 (5)	**<0.001**
Race				**<0.001**
Caucasian	34,552 (92.4%)	21,658 (92.1%)	12,894 (93%)	
African American	1389 (3.7%)	833 (3.5%)	556 (4%)	
Other	1434 (3.8%)	1014 (4.3%)	420 (3%)	
Smoking Status				**<0.001**
Former	19,060 (50.7%)	12,360 (52.2%)	6700 (48%)	
Current	18,567 (49.3%)	11,302 (47.8%)	7265 (52%)	
Pack-years	64.6 (23.6)	66.9 (25.1)	60.6 (20.4)	**<0.001**

Age, race, smoking status, and pack-years in the NLST cohort. Age and pack-years are given as mean (standard deviation). Numerical values as well as percentages are used for the categorical variables. Age and pack-year history were compared using independent *t*-tests and the other variables were compared using chi-squared tests. Significant *p*-values are bolded. n = number.

**Table 2:** Univariate and Multivariate Time-to-Event Cox Proportional Hazards Analyses for the NLST Cohort.

Variable	Univariate HR 95% (95% CI)	*p*-Value	Multivariate HR (95% CI)	*p*-Value
Age	1.076 (1.066–1.085)	**<0.001**	1.073 (1.063–1.082)	**<0.001**
Sex				
Female vs. Male	1.033 (0.939–1.137)	0.502	1.11 (1.007–1.222)	**0.035**
Pack-years	1.007 (1.006–1.009)	**<0.001**	1.006 (1.005–1.008)	**<0.001**

Univariate time-to-event analyses were performed to investigate the association between age, sex, and pack-year history with risk of developing lung cancer in the NLST cohort. All variables used in the univariate analysis were inputted into the multivariate analysis to account for confounding. Additionally, the interaction between sex and pack-years was inputted as an additional covariate. Age, sex, and pack-years were significant in the final analysis. The interaction between sex and pack-years was eliminated. Significant *p*-values are bolded. HR = Hazard Ratio. CI = Confidence Interval.

**Table 3:** Overall Patient Characteristics of the SEER Cohort as well as Differences Between Males and Females.

Variable	Overall n = 19,327	Male n = 9175 (47.5%)	Female n = 10,152 (52.5%)	*p*-Value
Age	70.5 (9.8)	70.4 (9.4)	70.9 (10.1)	0.086
Race				**0.005**
Caucasian	15,721 (81.3%)	7400 (80.7%)	8321 (82%)	
Non-Caucasian	1568 (8.1%)	738 (8%)	830 (8.2%)	
Unknown	2038 (10.5%)	1037 (11.3%)	1001 (9.9%)	
Grade				**<0.001**
Well/Moderately Well Differentiated	9175 (47.5%)	4131 (45%)	5044 (49.7%)	
Poorly Differentiated	4325 (22.4%)	2332 (25.4%)	1993 (19.6%)	
Unknown	5827 (30.1%)	2712 (29.6%)	3115 (30.7%)	
Histology				**<0.001**
Adenocarcinoma	12,528 (64.8%)	5254 (57.3%)	7274 (71.7%)	
Squamous Cell Carcinoma	6799 (35.2%)	3921 (42.7%)	2878 (28.3%)	

Age, race, tumor grade of first primary, and tumor histology of first primary in the SEER cohort. Age is given as mean (standard deviation). Numerical values as well as percentages are used for the categorical variables. Age was compared using independent *t*-test, and the other variables were compared using chi-squared tests. Significant *p*-values are bolded. n = number.

**Table 4:** Univariate and Multivariate Time-to-Event Cox Proportional Hazards Analyses for the SEER Cohort.

Variable	Univariate HR (95% CI)	*p*-Value	Multivariate HR (95% CI)	*p*-Value
Age	0.998 (0.993–1.002)	0.369	-	-
Sex				
Female vs. Male	**1.12 (1.022–1.228)**	**0.016**	**1.138 (1.02–1.269)**	**0.021**
Race				
Non-Caucasian vs. Caucasian	0.907 (0.764–1.078)	0.267	-	-
Grade				
Poorly Differentiated vs. Well/Moderately Well Differentiated	1.071 (0.959–1.196)	0.226	-	-
Histology				
Squamous Cell Carcinoma vs. Adenocarcinoma	**1.181 (1.073–1.3)**	**<0.001**	**1.171 (1.046–1.311)**	**0.006**

Univariate time-to-event analyses were performed to investigate the association between age, sex, race, grade of first primary lung cancer, and histology of first primary with risk of developing second primary lung cancer in the SEER cohort. All variables used in the univariate analysis were inputted into the multivariate analysis to account for confounding. Only sex and histology were retained in the multivariate analysis. Significant *p*-values are bolded. HR = Hazard Ratio. CI = Confidence Interval.

## Data Availability

Data for this study were extracted from two publicly available clinical databases: the National Lung Screening Trial (NLST) and Surveillance, Epidemiology, and End Results (SEER).
